# Integrative analysis of genes reveals endoplasmic reticulum stress-related immune responses involved in dilated cardiomyopathy with fibrosis

**DOI:** 10.1007/s10495-023-01871-z

**Published:** 2023-07-18

**Authors:** Wanpeng Li, Peiling Liu, Huilin Liu, Fuchun Zhang, Yicheng Fu

**Affiliations:** 1 Department of Cardiology, Gansu Provincial Hospital, Lanzhou, 730000, P.R. China; 2 Department of Rheumatology, First Affiliated Hospital of Zhengzhou University Zhengzhou, Henan, 450000, P.R. China; 3 Present Address: Department of Geriatrics, Peking University Third Hospital, Beijing, 100191, P.R China

**Keywords:** Endoplasmic reticulum stress, Dilated cardiomyopathy, Myocardial fibrosis, Bioinformatics, Immune cells

## Abstract

Endoplasmic reticulum (ER) stress has been implicated in the mechanisms underlying the fibrotic process in dilated cardiomyopathy (DCM) and results in disease exacerbation; however, the molecular details of this mechanism remain unclear. Through microarray and bioinformatic analyses, we explored genetic alterations in myocardial fibrosis (MF) and identified potential biomarkers related to ER stress. We integrated two public microarray datasets, including 19 DCM and 16 control samples, and comprehensively analyzed differential expression, biological functions, molecular interactions, and immune infiltration levels. The immune cell signatures suggest that inflammatory immune imbalance may promote MF progression. Both innate and adaptive immunity are involved in MF development, and T-cell subsets account for a considerable proportion of immune infiltration. The immune subtypes were further compared, and 103 differentially expressed ER stress-related genes were identified. These genes were mainly enriched in neuronal apoptosis, protein modification, oxidative stress reaction, glycolysis and gluconeogenesis, and NOD-like receptor signaling pathways. Furthermore, the 15 highest-scoring core genes were identified. Seven hub genes (*AK1*, *ARPC3*, *GSN*, *KPNA2*, *PARP1*, *PFKL*, and *PRKC*) might participate in immune-related mechanisms. Our results offer a new integrative view of the pathways and interaction networks of ER stress-related genes and provide guidance for developing novel therapeutic strategies for MF.

## Introduction

Dilated cardiomyopathy (DCM) is a structural cardiac disease characterized by ongoing heart failure, ventricular arrhythmia, and sudden cardiac death. Excessive myocardial fibrosis (MF) and neurohumoral activation of DCM could result in electrophysiological and structural remodeling, which further causes conduction system abnormalities and arrhythmogenicity[1]. Enhanced MF, characterized by fibrogenesis and excessive deposition of fibrous extracellular matrix proteins (ECM), is recognized as one of the key responses to DCM, from the development of myocardial remodeling and increased wall stress to progressive cardiac dysfunction [2]. Nevertheless, despite their high risk and severe morbidity, existing therapies seem to be complicated and ineffective [3].

Recently, progress has been made in identifying some biomarkers that reflect MF. These include galectin 3 (Gal3) [4], placental growth factor (PLGF) [5], cardiac ankyrin repeat protein (CARP) [6], platelet-derived growth factor (PDGF) [7], and suppression of tumorigenicity 2 (ST2) [8]. However, no clear relationships between these markers and drugs have been established, and they have not yet been applied to personal treatment. Because of these limitations, there is an urgent need to discover new biomarkers with clear mechanisms to delay, prevent or reverse MF and guide clinical treatment.

The mechanisms underlying MF remain unclear. Among the potential mechanisms that have recently gained significant attention, ER stress is an important etiology and pathological process in DCM-associated MF. Under constant stress conditions, such as oxidative stress, inflammation, gene defects, and cardiac ischemia, the unfolded protein response (UPR) pathways are activated and disturb the homeostasis of the ER [9]. ER stress could regulate cell apoptosis [10, 11], trigger secretory autophagy, and destroy target cells [12]. Recent studies have revealed the pathological role of ER stress in MF. For instance, the ER protein TXNDC5 is upregulated due to ER stress and promotes MF by facilitating extracellular matrix protein folding and cardiac fibroblast activation [1]. The overexpression of the transmembrane protein disintegrin and metalloproteinase 17 (ADAM17) could activate mouse cardiac fibroblasts (mCFs) by inhibiting the ATF6 branch of the ER stress response, further activating mitophagy and leading to MF [13]. In addition, ER stress could directly initiate inflammatory signaling pathways; cytokines and proinflammatory chemokines further trigger ER stress, and vice versa, finally resulting in an inflammatory cascade [14]. Persistent activation of the innate immune response could lead to chronic inflammatory processes that promote fibrotic deposition. Currently, no effective medical interventions for MF inhibit ER stress. Nevertheless, the suppression of ER stress is regarded as a promising therapy for MF; thus, there is a strong need to identify new pharmacological intervention targets to increase these opportunities for better treatments.

Bioinformatic analysis of microarray results has extended previous studies to provide meaningful gene information. This can be used to screen differentially expressed genes (DEGs), biological functional pathways, and promising targets for MF. Several previous studies have examined genetic alterations from different perspectives. One study investigated DEGs in 14 patients with DCM and 10 healthy controls. The results found that 11 key genes, including *CTGF, POSTN, CORIN*, and *FIGF*, are involved in ECM and cell-adhesion-related signaling pathways [15]. Another study investigated the tissue-specific expression profiles and epigenetic profiles of several genes critical for cardiac fibrosis, including *NLRP3*, *hsa-mir-223*, *MMP2*, and *MMP9*, and their enhancers contain hypomethylated transcription factor binding sites (TFBS) that might lead to the overexpression of genes and fibrotic phenotypes [16]. However, different chip platforms, statistical methods, and small sample sizes have contributed to some inconsistency in the findings of previous studies. Therefore, more in-depth studies are needed to identify reliable markers and new therapeutic targets for MF to overcome these potential inconsistencies.

To help address these issues, this study aimed to integrate microarray datasets and conduct an in-depth bioinformatics analysis to explore genetic changes in MF and screen for potential biomarkers related to ER stress. Specifically, we integrated two microarray datasets, including 19 DCM samples and 16 normal controls, to identify differentially expressed ER stress-related genes (DEERSRGs). Given the remarkable differences in the immune landscape between the subtypes of MF samples, we further conducted an extensive set of enrichment analyses focused on the biological functions and interaction networks of DEERSRGs. In addition, 7 hub genes including *AK1*, *ARPC3*, *GSN*, *KPNA2*, *PARP1*, *PFKL*, and *PRKC* were selected for participating in immune related mechanisms. This research will improve our understanding of the role of ER stress in DCM with fibrosis and will provide a potential target for the therapeutic strategies of MF.

## Materials and methods

### Data sources and processing

Reliable expression profiles of MF, GSE 3585 [17], and GSE 42,955 [18] were downloaded from the Gene Expression Omnibus (GEO) dataset using the Bioconductor package “GEOquery” with R software (version 4.0.4, http://r-project.org/) [19]. All samples were extracted from *Homo sapiens*, and the platforms were based on the GPL96 [HG-U133A] Affymetrix Human Genome U133A Array and GPL6244 [HuGene-1_0-st] Affymetrix Human Gene 1.0 ST Array [transcript (gene) version]. A total of 7 DCM and five healthy human myocardial samples from GSE42955 and 12 DCM and five healthy human myocardial samples from GSE42955 were included in the study. Raw data from GSE3585 and GSE42955 was deposited using the R “GEOquery” package [20]. ​The background correction and TMM normalization are used to obtain the gene expression matrix. TMM normalization(trimmed mean of M-values) method was used to normalize the RNA-seq data. It involves two key steps: library size adjustment and transcript-specific normalization. During the library size adjustment step, we ensure that the total read count per sample is normalized to a common library size. This adjustment accounts for differences in sequencing depth across samples, enabling valid comparisons. The TMM calculates a scaling factor to achieve this normalization while minimizing the effect of extreme outliers.For transcript-specific normalization, TMM aims to correct for biases related to transcript length and potential compositional effects. It accomplished this by estimating relative expression levels between samples based on log-fold changes (M-values) of individual genes. The trimmed mean was then calculated by excluding a specific percentage (usually 25%) of genes with the highest and lowest fold changes. This pruning helped to diminish the influence of genes with extreme fold changes that may not represent true biological variations.By applying TMM normalization, we aimed to reduce technical variations, enhance comparability between samples, and accurately identify differentially expressed genes (DEGs). Sequencing batch effects were removed using the “combat” function in R (sva) [21]. Plots of inter-sample correction were generated using the function “boxplot” in R.

### Assessment of immune cell infiltration

Deconvolution analysis was performed using the CIBERSORT algorithm, which employs a linear support vector regression model to evaluate relative subsets of RNA transcripts [22]. The gene expression matrix was further analyzed using the CIBERSORT algorithm to estimate the 22 infiltrating immune cell subsets in the samples. The samples were filtered with an adjusted *P*-value of ≤ 0.05, and the output of the MOABS algorithm and immune cell infiltration matrix was computed. The correlations among the 22 infiltrating immune cells were calculated and visualized using the R function “corr plot” [23]. Single-sample GSEA analysis (ssGSEA) was performed using the R package “GSVA” [24], and the correlation between the samples was visualized using the R package “pheatmap” (https://CRAN.R-project.org/package=pheatmap) [24].

### Construction of immune subtypes and screening for DEGs

The immune subtypes of MF were calculated with the R extension packages “ConsensusClusterPlus” [25] and “Rtsne” [26], and the visualization was implemented using the R package “ggplot2” [27]. To identify the DEGs, volcano plots were visualized using the “ggplot2” R package, and heatmaps were generated using the “pheatmap” R package. We defined significant DEGs with an adjusted *P*-value < 0.05 and |log2FC| > 0.1.

### Functional enrichment analysis

The Gene ontology (GO) and Kyoto Encyclopedia of Genes and Genomes (KEGG) enrichment analyses and Disease Ontology (DO) analysis were conducted for gene intersections using the R package “clusterProfiler” [28]. The GO enrichment analysis was performed using the Metascape website [29] and was visualized on DisGeNET [30] using disease ontology analysis. The gene expression matrix was then intersected with ER stress-related genes, and GSEA was performed [31]. The gene set “c2.cp.kegg.v7.0.entrez.gmt” was chosen as a reference. A false discovery rate (FDR) < 0.25 with *P* < 0.05 was considered significant. GSVA was implemented using the R package “GSVA,” where *P* < 0.05 was considered significantly enriched.

### Protein-protein interaction (PPI) analysis and identification of hub genes

PPI networks were extracted from the STRING database (https://string-db.org/) [32] and imported into Cytoscape 3.8.2 for visualization [33]. Hub genes were screened simultaneously using the “Cytohubba” plug-in [34]. The correlation between the hub genes and different immune cells and the association of immune subtypes and immune cellular features were calculated and drawn using the “ggpubr” package (https://CRAN.R-project.org/package=ggpubr).

### Network analysis of key genes

By selecting TF-gene Interaction in Gene Regulatory Networks (GRN), the Network Analyst platform (https://www.networkanalyst.ca/) was used to analyze the interaction between the hub genes and potential transcription factors (TFs) based on the JASPAR database [35]. We then used the miRTarBase database [36] and chose gene-miRNA interactions to identify the interaction between the hub genes and potential miRNAs. Finally, we chose “Protein-drug Interactions” in “Diseases, drugs & chemicals” and assessed the interactions between the hub genes and potential drug effects based on DrugBank database analysis [37].

### Statistical analysis

All statistical analyses were performed using R statistical software (version 4.0.4, http://r-project.org/). The independent *t*-test was used for normally distributed continuous data, and the Mann-Whitney *U* test was used for non-normally distributed continuous data. All statistical tests were two-sided, and *P* ≤ 0.05 was considered statistically significant.

## Results

### Data pre-processing

**Fig. 1:**
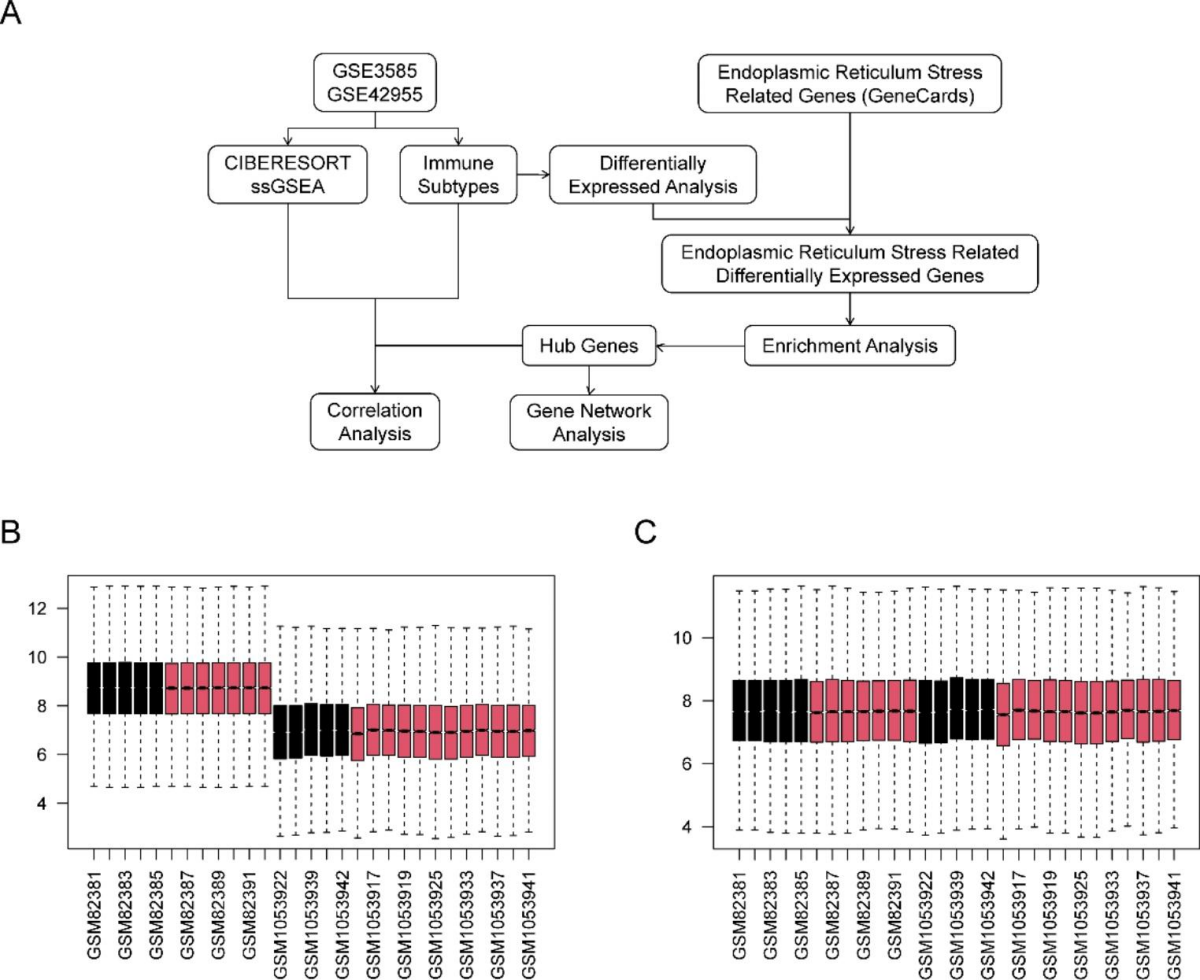
Flow chart and boxplots of consolidated datasets before and after removing batch effects. **A** Flow chart of methodologies applied in the current study. **B** Boxplots of consolidated datasets (GSE3585 and GSE42955) before removing the batch effects. **C** Boxplots of consolidated datasets (GSE3585 and GSE42955) after removing the batch effects

The microarray data analysis used in this study is shown schematically in Fig. 1A; Table 1. The GSE3585 and GSE42955 datasets were downloaded from the GEO repository and normalized within R. We subsequently merged the two datasets and confirmed that the batch effect between them was removed. The gene expression signatures before and after batch-effect correction are displayed in the form of boxplots in Fig. 1B C, respectively.

**Table 1:** GEO microarray data used to identify altered DEERSRGs in MF samples

	Platform	Number of test	Number of control	Country	Year	Author
GSE3585	GPL96	7 fibrosis	5 control	Germany	2006	Ruprecht Kuner
GSE42955	GPL6244	12 fibrosis	5 control	Spain	2013	Maria Micaela Molina Navarro

### Differential expression analysis of Immune Cell Infiltration

**Fig. 2:**
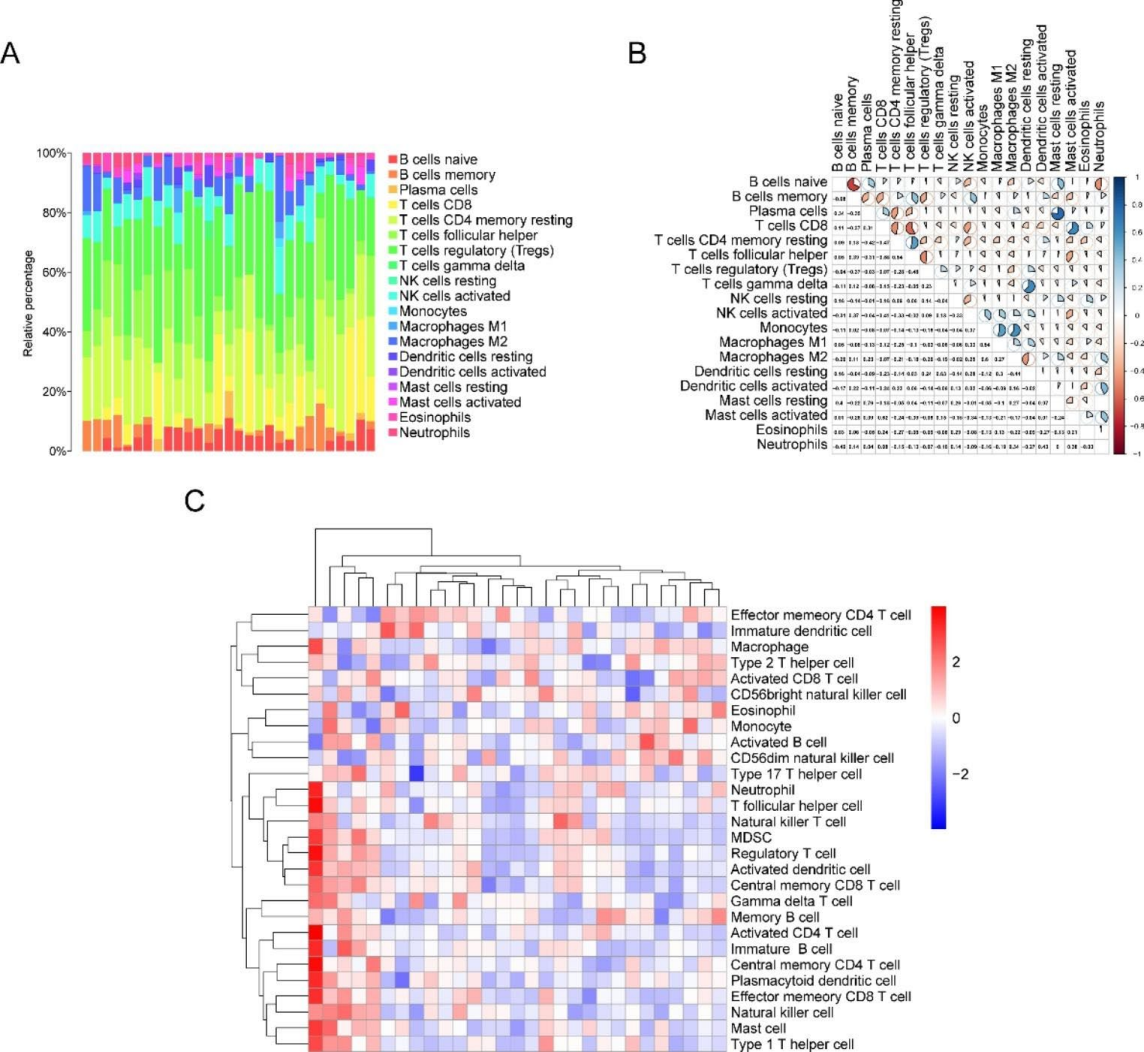
Assessment and visualization of immune cell infiltrates. **A** Bar plot of overall immune cell proportions. **B** Correlation heatmap depicting the correlations between infiltrating immune cells in MF tissues. The numbers in the plots represent the Pearson’s correlation coefficient. **C** Correlation between sample infiltration levels and 28 immune cell types in MF tissues. The blue shading denotes negative genetic correlations, and the red shading denotes positive correlations, with gradations of color intensity reflecting an increasing strength in the correlation. MF: Myocardial Fibrosis

We estimated the level of immune cell infiltration in patients with MF using the CIBERSORT algorithm. Among them, T-cell subsets represented the dominant proportion of infiltrating immune cells (Fig. 2A). The correlation coefficient analysis revealed a significant correlation between the levels of immune cell infiltration (Fig. 2B). The ssGSEA of the per-sample infiltration levels of 28 immune cell types revealed the enrichment of diverse immune cell populations in a subset of MF patients (Fig. 2C).

### Construction of subtypes with different immune signatures, screening of differential genes, and visualization

**Fig. 3:**
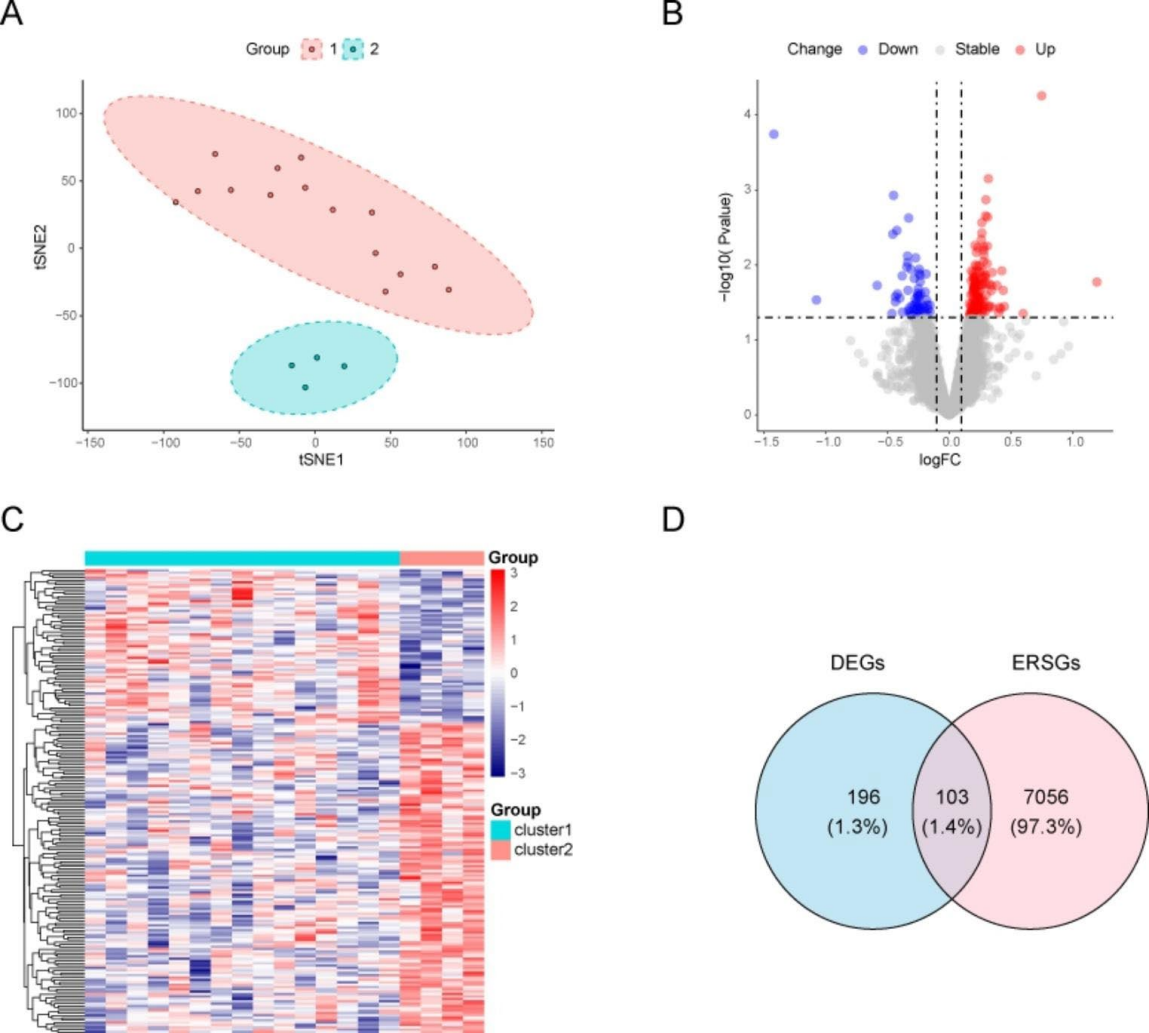
Construction of characteristic immune subtypes, screening of differential genes, and visualization. **A** Construction of subtypes with different immune signatures. **B** Differential gene expression between subtypes is shown in a volcano map. Here, red represents upregulated genes and blue represents downregulated genes. **C** Heat map depicting a series of differential genes between two subtypes. **D** Venn diagram demonstrating the intersections of DEGs between ERSRGs. DEGs: ERSRGs: ER stress-related genes

Clustering analysis was conducted based on immunohistochemical characteristics. Immunohistochemistry is a laboratory method used to visualize and analyze the presence, distribution, and localization of specific proteins or antigens in tissue samples. During immunohistochemical analysis, the tissue sample is first fixed and embedded in a solid medium (usually paraffin). The tissue is then sliced and mounted on a slide. These slices are then subjected to a series of steps, including dewaxing, antigen repair, sealing, and incubation with primary and secondary antibodies. The primary antibodies used in immunohistochemistry are specific to the protein or antigen of interest. When a primary antibody binds to its target protein, it can be visualized using a detection system such as colored enzyme substrates or fluorescent dyes. The resulting staining pattern provides information about the presence, abundance, and cell localization of the target protein in the tissue sample. These immunohistochemical features are used to group or classify tissue samples based on similarities or differences in protein expression profiles. According to immunohistochemical characteristics, the MF patients were divided into two subgroups—“cluster1” and “cluster2”—with notable differences (Fig. 3A).To test the differences between gene expression levels in patients with MF, we performed differential gene expression analysis on these subtypes. We identified 196 DEGs, including 131 upregulated and 65 downregulated genes. A volcanic map depicting differential gene expression between the subtypes is shown in Fig. 3b, and a heat map depicting a sequence of differential genes between the two subtypes is shown in Fig. 3C. Furthermore, the overlap between the DEGs and ER stress-related genes (ERSRGs) was calculated, and 103 genes DEERSRGs were identified (Fig. 3D).

### Functional enrichment analysis

**Fig. 4:**
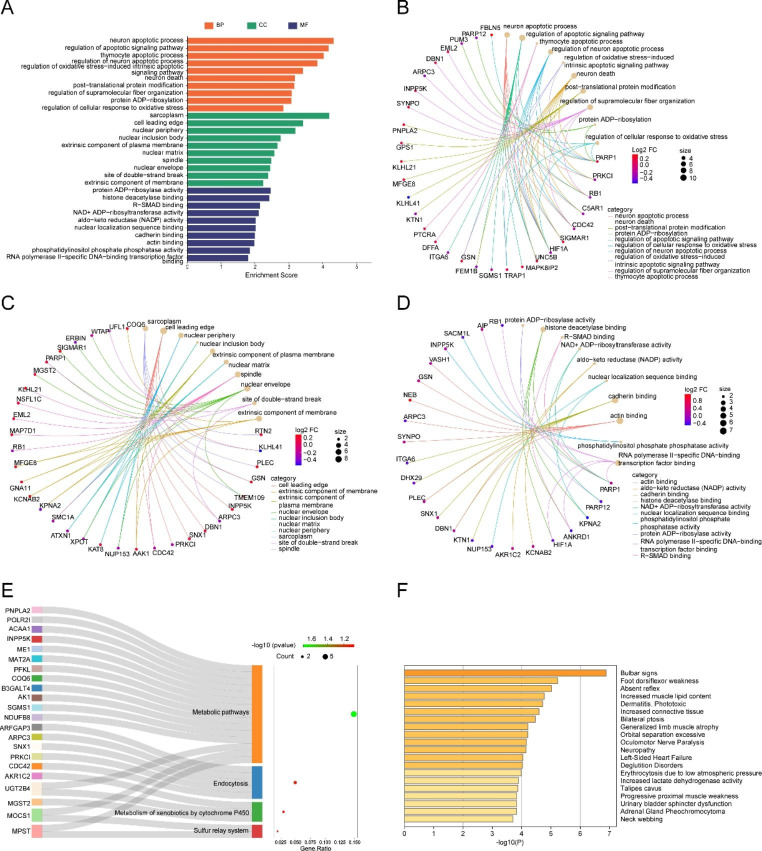
Functional enrichment analyses of the genes modified by DEERSRGs. **A** GO analysis revealed the most enriched categories for biological processes (BP), cellular components (CC), and molecular functions (MF). **B** A meshwork of GO clusters for the BP. **C** A meshwork of GO clusters for the CC. **D** A meshwork of GO clusters for the MF. **E** Sankey diagram of the significant KEGG pathways. **F** Histogram of the DO analysis. BP: Biological Processes; CC: Cellular Components; MF: Molecular Functions; GO: Gene Ontology; KEGG: Kyoto Encyclopedia of Genes and Genomes; DO: Disease Ontology

GO, KEGG, and Disease Ontology (DO) enrichment analyses were performed to explore the biological classification of the DEERSRGs, the results of which are shown in Fig. 4. Figure 4 A shows the 10 highest-ranking GO terms, i.e., biological processes (BPs), cellular component (CC), molecular function (MF), ontology, neuron apoptotic process, sarcoplasm, and protein ADP-ribosylase activity, that are most closely related. These are mainly involved in important BPs such as the “neuron apoptotic process,” “apoptotic signaling pathway,” “neuron death,” “post-translational protein regulation,” and “regulation of supramolecular fiber organization” (Fig. 4B), indicating a close association between DEERSRGs and the apoptosis and fibrotic process. The highly enriched GO terms for the cellular component were “sarcoplasm,” “cell leading edge,” “nuclear periphery,” and “extrinsic component of membrane” (Fig. 4C). Regarding MFs, “protein ADP ribosylase activity,” “histone deacetlylase binding,” “cadherin binding,” and “actin binding transcription factor binding” were significantly enriched (Fig. 4D). The KEGG pathway analysis demonstrated that the DEERSRGs were mainly enriched in “metabolic pathways,” “endocytosis,” “metabolism of xenobiotics by cytochrome P450,” and the “sulfur relay system” (Fig. 4E). Detailed enrichment results are presented in Table 2. DO is an open-source ontology for the integration of human disease data and uses semantic similarity to explore disease similarity. Our results of the DO analysis showed that DEERSRGs also had a major impact on diseases such as bulbar signs, foot dorsiflexor weakness, and absent reflexes (Fig. 4F).

**Table 2:** Significant GO terms and KEGG pathway enrichment analysis of the modified DEERSRGs in MF.

ONTOLOGY	ID	Description	GeneRatio	P.Value	Count
BP	GO:0051402	neuron apoptotic process	8/100	4.80123E-05	8
BP	GO:2,001,233	regulation of apoptotic signaling pathway	10/100	6.7921E-05	10
BP	GO:0070242	thymocyte apoptotic process	3/100	9.30975E-05	3
BP	GO:0043523	regulation of neuron apoptotic process	7/100	0.000143615	7
BP	GO:1,902,175	regulation of oxidative stress-induced intrinsic apoptotic signaling pathway	3/100	0.000385337	3
BP	GO:0070997	neuron death	8/100	0.000658347	8
BP	GO:0043687	post-translational protein modification	8/100	0.00069493	8
BP	GO:1,902,903	regulation of supramolecular fiber organization	8/100	0.000828909	8
BP	GO:0006471	protein ADP-ribosylation	3/100	0.000836142	3
BP	GO:1,900,407	regulation of cellular response to oxidative stress	4/100	0.001453007	4
CC	GO:0016528	sarcoplasm	5/103	6.455E-05	5
CC	GO:0031252	cell leading edge	9/103	0.00038364	9
CC	GO:0034399	nuclear periphery	5/103	0.000646646	5
CC	GO:0042405	nuclear inclusion body	2/103	0.001751292	2
CC	GO:0019897	extrinsic component of plasma membrane	5/103	0.00216984	5
CC	GO:0016363	nuclear matrix	4/103	0.00264274	4
CC	GO:0005819	spindle	7/103	0.003283326	7
CC	GO:0005635	nuclear envelope	8/103	0.003533679	8
CC	GO:0035861	site of double-strand break	3/103	0.004090612	3
CC	GO:0019898	extrinsic component of membrane	6/103	0.005619794	6
MF	GO:1,990,404	protein ADP-ribosylase activity	2/101	0.003422239	2
MF	GO:0042826	histone deacetylase binding	4/101	0.003745662	4
MF	GO:0070412	R-SMAD binding	2/101	0.00703676	2
MF	GO:0003950	NAD + ADP-ribosyltransferase activity	2/101	0.007649096	2
MF	GO:0004033	aldo-keto reductase (NADP) activity	2/101	0.009624063	2
MF	GO:0008139	nuclear localization sequence binding	2/101	0.009624063	2
MF	GO:0045296	cadherin binding	6/101	0.010098042	6
MF	GO:0003779	actin binding	7/101	0.010462289	7
MF	GO:0052866	phosphatidylinositol phosphate phosphatase activity	2/101	0.014171103	2
MF	GO:0061629	RNA polymerase II-specific DNA-binding transcription factor binding	5/101	0.016050006	5
KEGG	hsa01100	Metabolic pathways	15/101	0.020119782	15
KEGG	hsa04122	Sulfur relay system	2/101	0.063564379	2
KEGG	hsa04144	Endocytosis	5/101	0.071527369	5
KEGG	hsa00980	Metabolism of xenobiotics by cytochrome P450	3/101	0.084058908	3

Abbreviations: BP, biological process; MF, molecular function; CC, cellular component; GO, Gene Ontology; KEGG, Kyoto Encyclopedia of Genes and Genomes;

### GSEA and GSVA

**Fig. 5:**
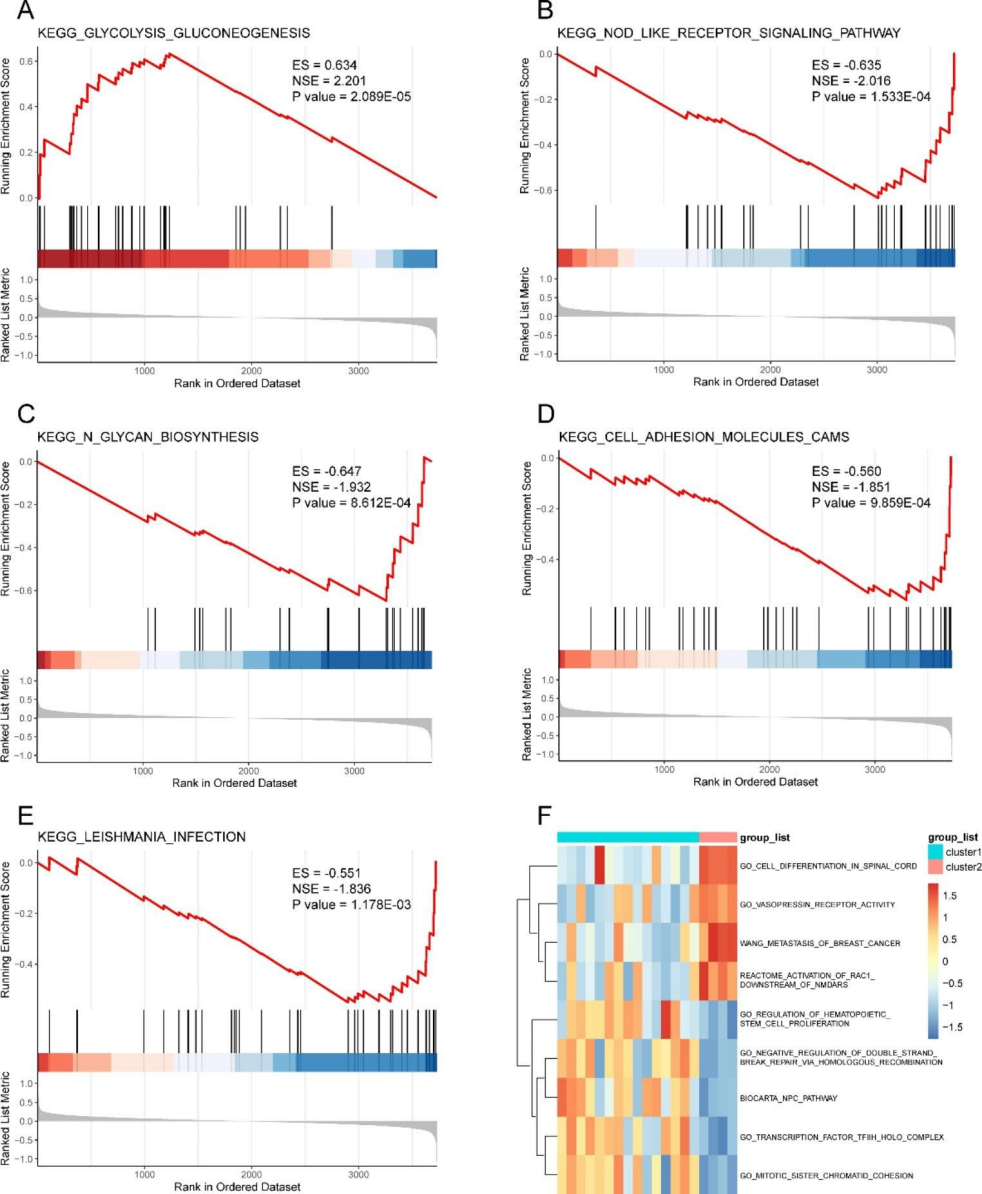
GSEA and GSVA enrichment analysis. **A–E** Five pathways obtained via GSEA enrichment analysis. **F** Heat maps of nine pathways obtained via GSVA enrichment analysis. GSEA: Gene Set Enrichment Analysis; GSVA: Gene Set Variation Analysis

A total of 3,731 genes were obtained from the intersection of all differentially changed genes of patients and ERSRGs, and we subsequently performed GESA (see Table 3) and GSVA (see Table 4) for these genes. The GSEA results indicated that the DEGs were significantly associated with five gene set pathways, namely “glycolysis and gluconeogenesis,” “cell adhesion molecules (CAMs),” “NOD-like receptor signaling pathway,” “N-glycan biosynthesis,” and “leishmania infection” (Fig. 5A–E). The GSVA results suggested that differential genes might participate in the regulation of nine signaling pathways.

**Table 3:** Results of GSEA analysis

ID	enrichmentScore	NES	P.Value	P.adjust	qvalues
KEGG_GLYCOLYSIS_GLUCONEOGENESIS	0.633571309	2.201461516	2.09E-05	0.002862248	0.00263903
KEGG_NOD_LIKE_RECEPTOR_SIGNALING_PATHWAY	-0.634560202	-2.016369297	0.000153308	0.010501608	0.00968262
KEGG_N_GLYCAN_BIOSYNTHESIS	-0.647374733	-1.931555731	0.000861228	0.032271182	0.029754451
KEGG_CELL_ADHESION_MOLECULES_CAMS	-0.560157171	-1.851001168	0.000985905	0.032271182	0.029754451
KEGG_LEISHMANIA_INFECTION	-0.550999577	-1.8357674	0.00117778	0.032271182	0.029754451

**Table 4:** Results of gene set enrichment analysis (GSEA)

ID	enrichmentScore	NES	P.Value	P.adjust	qvalues
KEGG_GLYCOLYSIS_GLUCONEOGENESIS	0.633571309	2.201461516	2.09E-05	0.002862248	0.00263903
KEGG_NOD_LIKE_RECEPTOR_SIGNALING_PATHWAY	-0.634560202	-2.016369297	0.000153308	0.010501608	0.00968262
KEGG_N_GLYCAN_BIOSYNTHESIS	-0.647374733	-1.931555731	0.000861228	0.032271182	0.029754451
KEGG_CELL_ADHESION_MOLECULES_CAMS	-0.560157171	-1.851001168	0.000985905	0.032271182	0.029754451
KEGG_LEISHMANIA_INFECTION	-0.550999577	-1.8357674	0.00117778	0.032271182	0.029754451

### PPI Network Analysis and Hub Gene Selection

**Fig. 6:**
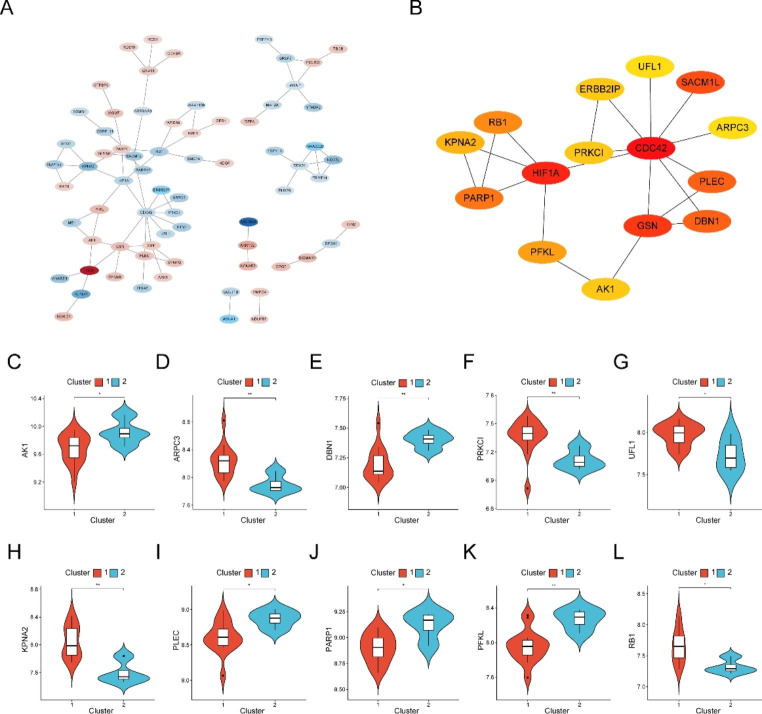
Protein-protein interaction (PPI) network analysis and hub gene screening. **A** The STRING database was used to generate the PPI interaction network of 103 DEERSRGs. **B** Hub genes screened using cytoHubba plug-in. **C** Differential expression analysis of hub genes between two immune subtypes. DEERSRGs: Differentially Expressed ER Stress-related Genes; PPI: Protein-protein Interaction

We used the STRING database to mine the key genes and imported DEERSRGs into the database to form a PPI network (Fig. 6A). The “cytoHubba” plug-in (https://apps.cytoscape.org/apps/cytohubba) [34] was used to obtain 15 hub genes, namely *GSN*, *HIF1A*, *ERBB2IP*, *RB1*, *SACM1L*, *ARPC3*, *PARP1*, *CDC42*, *KPNA2*, *PRKCI*, *DBN1*, *PLEC*, *AK1*, *PFKL*, and *UFL1* (Fig. 6B). Subsequently, we found that the expression levels of *AK1*, *DBN1*, *PLEC*, *PARP1*, and *PFKL* in cluster2 were higher than that in cluster1, and the expression levels of *ARPC3*, *PRKCI*, *UFL1*, *KPNA2*, and *RB1* in cluster1 were higher than that in cluster2 (Fig. 6C–L).

### Correlation analysis of hub genes and Immune Infiltration

**Fig. 7:**
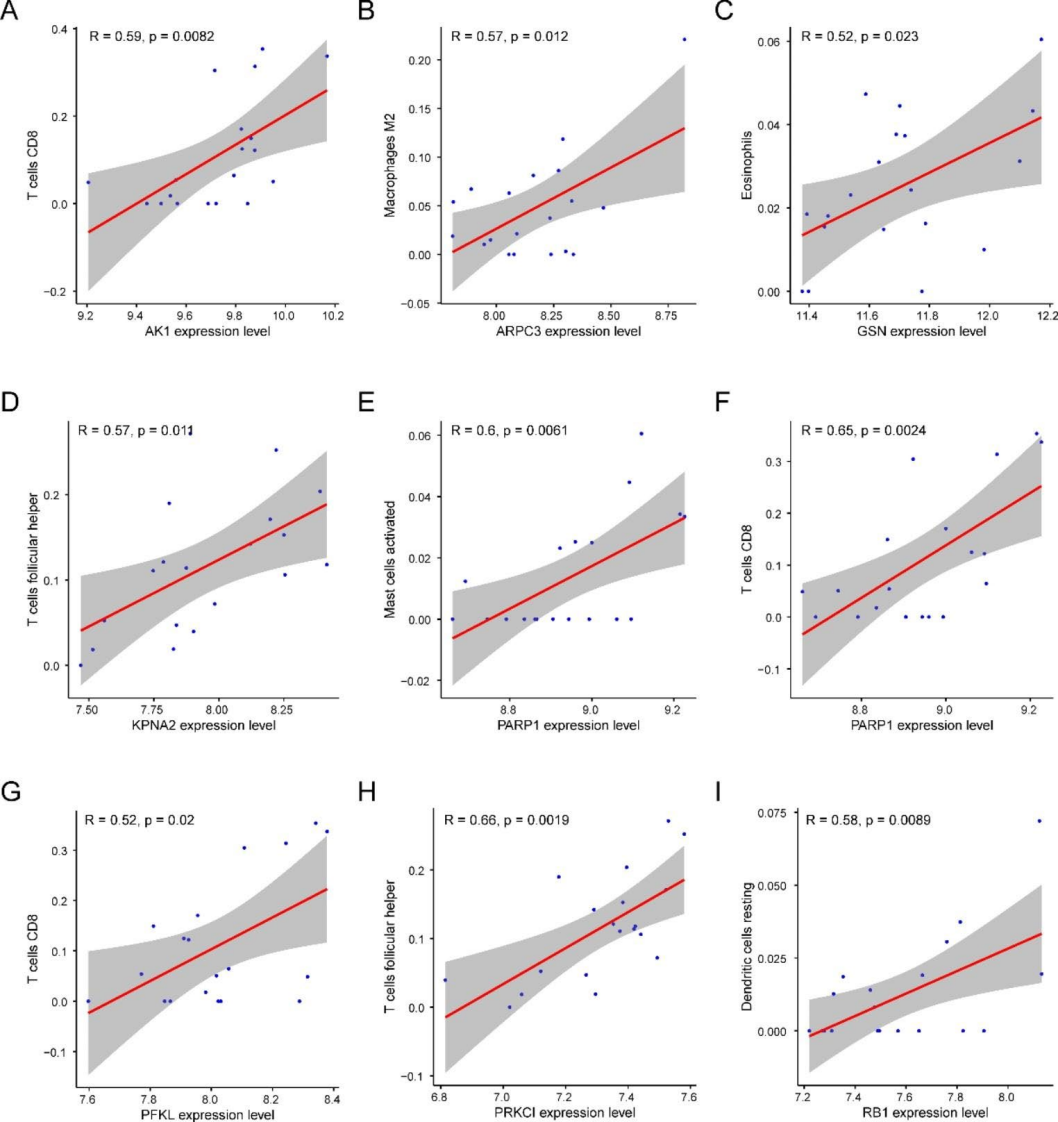
Correlation between hub genes and immune infiltrating cells. **A–I** Scatter plots of the correlation analysis between hub genes and immune cell types

The correlative relationships between ER stress-related hub genes and immune cell infiltration levels in MF were investigated using a preliminary analysis, from which we found the following significant associations: *AK1* was associated with CD8^+^ T cells, *ARPC3* with macrophage M2 cells, *GSN* with eosinophils cells, *KPNA2* with T follicular helper cells, *PARP1* with activated mast cells and CD8^+^ T cells, *PFKL* with CD8^+^ T cells, *PRKCI* with T follicular helper cells, and *RB1* with dendritic resting cells (R ≥ 0.5, *P* < 0.05) (Fig. 7A–I).

### Correlation between Immune infiltrating cells and Immune Subtypes

**Fig. 8:**
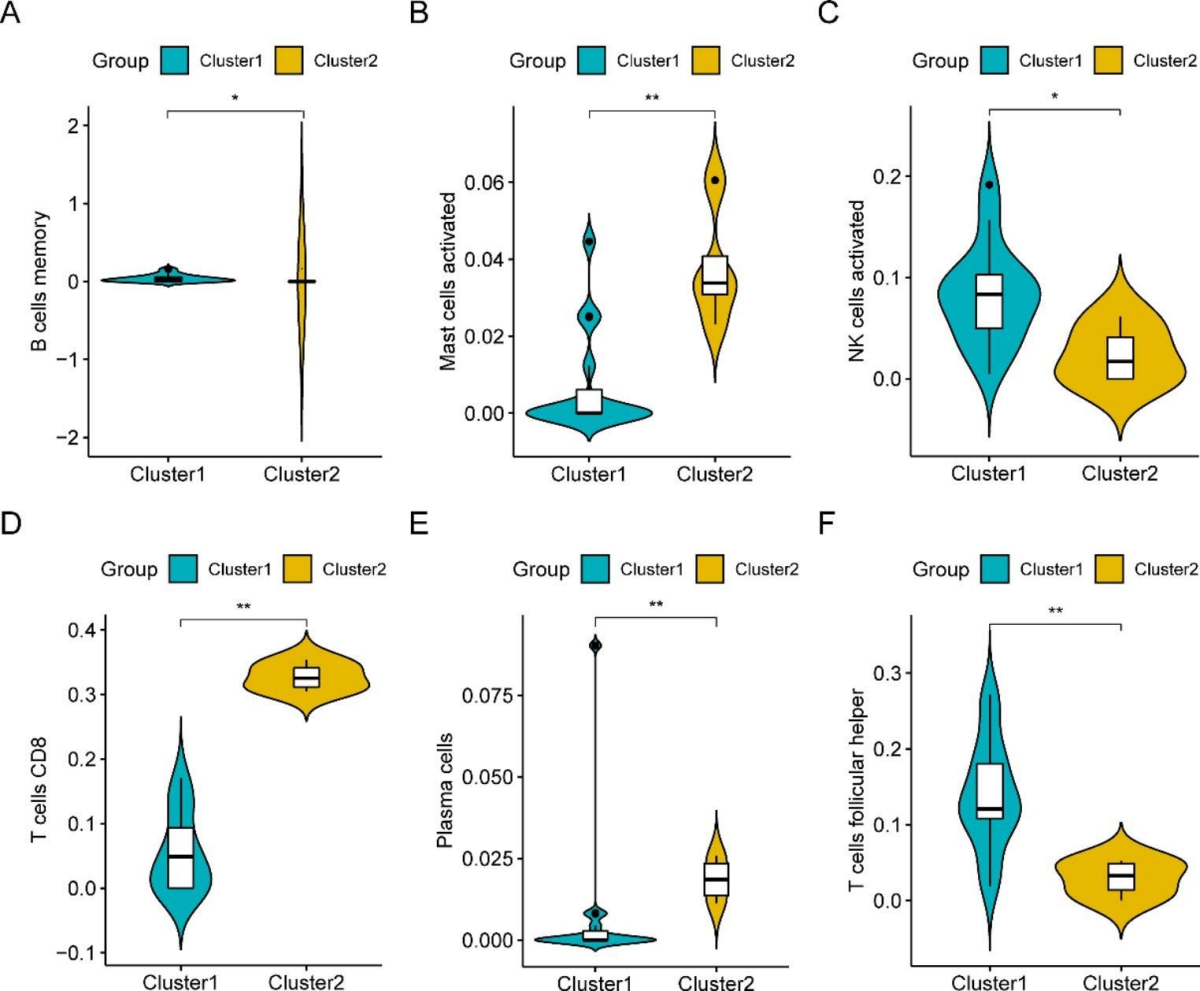
Correlation between immune infiltrating cells and immune subtypes. **A–F** Violin plots of differential expression analysis between the six immune cell types and subtypes associated with different immune signatures

We also found significant differences in infiltration levels between the two immune subtypes. The extent of infiltration of memory B cells, activated NK cells, and follicular helper T cells was higher in cluster1 (Fig. 8A, C, and F), while the extent of infiltration of activated mast cells, plasma cells, and CD8^+^ T cells appeared to be higher in cluster 2 (Fig. 8B, D, and E).

### Construction of PPI and ceRNA Networks

**Fig. 9:**
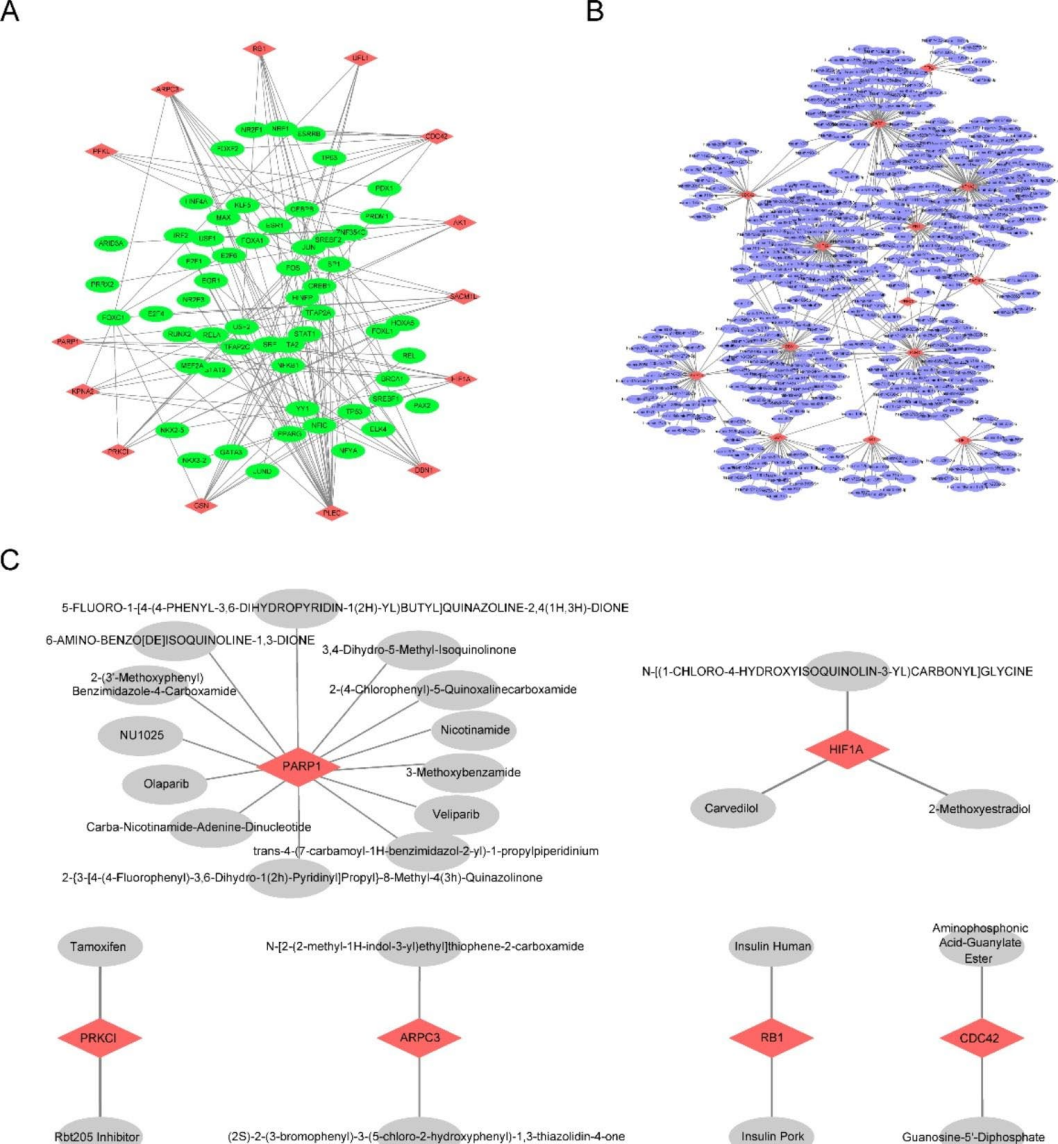
Gene network analysis. **A** Transcriptional network relationships with hub genes, where red represents hub genes and green represents transcription factors. **B** Network relationships of hub genes with miRNA, where red represents hub genes and purple represents miRNAs. **C** Network relationships between hub genes and drugs, where red represents hub genes and gray represents drugs

Based on the hub genes obtained from our previous study, we further analyzed their mutual regulatory relationships with other small molecules, including regulatory network relationships with transcription factors (TFs) (Fig. 9A), network relationships with miRNAs (Fig. 9B), and network relationships of the interactions between hub genes *PARP1*, *PRKC1*, *ARPC3*, *HIF1A*, *RB1*, and *CDC42* and different small molecules and drugs (Fig. 9C).

## Discussion

MF is correlated with elevated mortality in DCM, which is one of the most common cardiomyopathies globally and is directly associated with sudden cardiac death, heart failure, and life-threatening arrhythmia [38]. However, there are no clinically effective methods to inhibit MF progression. Thus, identifying novel and effective molecular therapeutic targets is of critical importance. Accumulating evidence suggests that ER stress is a key etiological component in the development and progression of MF [39]. Therefore, we conducted a comprehensive bioinformatics analysis of two microarray datasets (GSE3585 and GSE42955), including 19 DCM cardiac tissue and 10 normal cardiac tissue samples.

We observed significant differences in the total immune infiltration in patients with MF. The ssGSEA showed a higher level of T-cell infiltration in the MF samples than in the normal samples. We subdivided the samples into two immune clusters and identified 103 DEERSRGs for further analysis. The GO enrichment analysis showed that the DEERSRGs were primarily enriched in regulating neuronal apoptotic processes, sarcoplasm, and protein ADP-ribosylase activity. The KEGG enrichment analysis of the DEGs revealed that the DEERSRGs might be involved in pathways related to metabolism, endocytosis, cytochrome P450, and the sulfur relay system. Using GSEA/GSVA, we identified markedly enriched pathways, such as glycolysis/gluconeogenesis, glycan biosynthesis, and NOD-like receptor signaling, in the MF samples. Based on the PPI network, 15 hub genes with expression levels significantly correlated with MF pathogenesis were selected. We showed that the expression of ERSRGs not only varied greatly between the two immune clusters but was also correlated with the levels of different infiltrating immune cells. Subsequently, relationships between the hub genes and small-molecule compounds, TFs, miRNAs, and drugs were illustrated. Thus, this research improves our understanding of the role of ER stress in MF, and the predicted genes in these datasets serve as promising therapeutic targets or prognostic biomarkers.

First, we observed a close correlation between MF tissue and immune infiltration, which uncovered immunomodulatory mechanisms of fibrosis mediated by the recruited immune cell subsets. Second, we found a large proportion of T cell-rich infiltrates, which might have important biological effects on the pathogenesis of MF. The ssGSEA indicated that both the innate and adaptive immune systems are involved in the occurrence of MF, which is consistent with previous research findings [40]. In the classical pathogenic process of DCM, the innate immune response is activated first, followed by an adaptive immune response and a chronic phase that might persist for several months or years, which causes MF and remodeling, culminating in DCM [41]. Immune cell imbalance is a driving force in the promotion of MF progression. T cells are the most important immune-competent cells for cell-mediated immune response, and nearly 50% of patients with DCM show cardiac T-cell infiltrates [42]. T lymphocytes express multiple CAMs involved in the development of MF [43], and T cell abundance is closely linked to the degree of MF in DCM patients [44]. Therefore, for the analysis of differential genes, the gene expression profiles included in our study were subsequently divided into subtypes according to their immune cell signatures.

ER stress contributes to the pathogenesis of MF and disease progression in cardiac remodeling [1]. Pathological ER stress reflects an imbalance in ER homeostasis, and the UPR attenuation reaction from ER stress might inhibit fibrosis to some extent [45]. Therefore, investigating the molecular therapeutic targets involved in the pathogenesis of MF in detail is essential to improve the clinical outcomes of DCM. In the present study, we identified 196 DEGs, of which 131 were upregulated, and 65 were downregulated. We then screened 103 DEERSRGs for which PPI networks were constructed, which revealed the following top 15 genes as the most significant hub genes: *GSN*, *HIF-1α*, *ErbB2IP*, *RB1*, *SACM1L*, *ARPC3*, *PARP1*, *CDC42*, *KPNA2*, *PRKCI*, *DBN1*, *PLEC*, *AK1*, *PFKL*, and *UFL1*. These hub genes were found to be associated with ER stress in this study and were also previously reported to be closely related to MF. For example, the *GSN* gene encodes the gelsolin protein, which is involved in cytoskeletal maintenance. Downregulation of the *GSN* gene might lead to MF [46]. Hypoxia-inducible factor 1α (*HIF-1α*) is a key mediator of hypoxia-induced MF; it can regulate the production of reactive oxygen species in the mitochondria and cause cardiac fibroblast proliferation. Fibroblasts tend to be more hypoxic than other cardiac interstitial cells and, therefore, can express more HIF-1α and exhibit increased glycolysis [47]. The *ErbB2* gene has been found to play a key role in cardiomyocyte proliferation and in inducing DCM [48]. Poly ADP-ribose polymerase 1 (PARP1) accelerates MF through NAD-dependent mTOR activation. The *PARP1* gene can regulate autophagy, cause TGF-β1-induced proliferation, and affect the activity of cardiac fibroblasts[9]. It is worth mentioning that the distribution of hub gene expression in MF subtypes was also notably different. Our results also revealed that the expression of the following eight hub genes was closely related to the levels of specific innate immune cell types: *AK1, ARPC3, GSN, KPNA2, PARP1, PFKL, PRKCI*, and *RB1*. These potential links between overlapping hub genes might provide new perspectives for developing new treatment strategies.

The GO analysis confirmed that DEERSRGs had significantly higher expression in the BP category involving neuronal apoptotic processes, apoptotic signaling pathways, and neuronal death. We found that the major significantly enriched pathways were closely associated with cell apoptosis and death. ER stress and the UPR inhibit cellular protein synthesis and degradation of misfolded proteins, ultimately inducing apoptosis, necrosis, and autophagy in cardiomyocytes. Multiple studies have confirmed that augmentation of ER stress promotes neuronal death and apoptosis [49, 50]. In addition, many studies have demonstrated that ER stress has a similar effect on cardiovascular diseases. For example, one study found that in patients with DCM with *FBXO32* (MAFbx, Atrogin-1) mutations, UPR activity was reduced as well as the expression of target genes, and CHOP TFs were upregulated, which can lead to CHOP-associated apoptosis of cardiomyocytes [51]. In addition, the deletion of *PKA2*, which encodes a stress-responsive kinase located near the ER membrane, can also lead to poor ER stress and cardiac cell death [52]. Furthermore, we identified some unstudied BPs, such as post-translational protein modification, oxidative stress, and fiber organization, as well as related molecular functions and cellular localization, which might help better understand the development and progression of MF.

The traditional view holds that MF is mainly involved in activating the renin-angiotensin system, inflammatory signaling, and ECM remodeling [16]. Previous microarray analysis of the microRNA datasets GSE3585 and GSE42955 showed that MF-associated DEGs were upregulated in the ECM–receptor interaction and focal adhesion [15]. Our KEGG pathway analysis revealed several new candidate biological pathways implicated in ER stress-induced MF, including the metabolism, endocytosis, cytochrome P450, and sulfur relay system pathways. ​Our results are consistent with those of several other studies related to ER stress and fibrosis.​ For example, spermine metabolic disorders can cause MF and ER stress in the myocardial tissue of DCM rats via the Wnt/β-catenin signaling pathway [53]. ER stress can also enhance caveolin-1-mediated endocytosis in calreticulin-knockout mouse embryonic fibroblast cells [54]. Exogenous drug metabolism by cytochrome P450 can also trigger ER stress and activate the IRE1α-TRAF2-NF-κB signaling pathway [55]. Moreover, we used DO analysis to construct an approximate visualization of new gene–disease associations. Our results showed associations highlighted in neurodegenerative disorders, neuromuscular disorders, and left heart failure caused by cardiac injury and fibrosis. These findings reveal a possible path through which DEERSRGs interact and might provide useful directions for future research.

The GSEA and GSVA revealed the most relevant pathways associated with DEERSRGs, including glycolysis/gluconeogenesis, glycan biosynthesis, NOD-like receptor signaling pathway, and CAM-associated pathway. Altered cardiac glucose metabolism is an early feature of cardiomyopathy and a crucial cause of cardiac remodeling, fibrosis, and myocardial dysfunction. Advanced glycation end products can stimulate collagen deposition by promoting collagen cross-linking, which leads to increased MF [56]. NOD-like receptors can recognize danger signals, recruit immune cells to the region of myocardial injury areas, and activate an inflammatory response. The activation of NOD1 receptors induces apoptosis and activates the TGF-β pathway in cardiac fibroblasts [57]. Intercellular and vascular cell adhesion molecules have been postulated to affect cardiac oxidative stress, remodeling, and fibrosis [43]. Furthermore, the analysis of microarrays for MF from other mRNA datasets indicates that core genes participate in pathways associated with biological adhesion [15]. These findings are important for interpreting our gene enrichment analysis results.

We further determined the regulatory molecular signatures and potential candidate drug signatures of the DEERSRGs and mapped the distribution of TFs and miRNAs corresponding to each hub gene. We identified many new TFs that can bind to the promoters of several crucial regulators in differentiated myocardial cells that remain unstudied. Previous studies have reported several TFs that have a marked impact on disease progressions, such as SRF, GATA-4, HAND2, TBX-20, MEF2C, FOXO, and HEY2 [58–60]. In addition, we performed an in-depth analysis of the relationships between the DEERSRGs and the miRNA networks. Previous studies have found several microRNAs, such as miR-5571-5p, miR-26, and miR-30, to be closely related to the excessive accumulation of ECM in MF [61, 62]. miR-185 inhibits the activity of B cells, which are involved in MF and myocyte injuries in DCM [63]. Finally, we constructed a small-molecule drug–target gene interaction network of the core genes. This network can be used to screen the affinity between compounds and gene targets, and insights into this relationship would facilitate gene-targeted drug discovery and assist in identifying new drug-binding sites. Furthermore, the molecular basis for specific gene regulation and drug sensitivity provides a reference for exploring specific DEERSRGs as potential therapeutic targets.

This study has some limitations that need to be acknowledged. First, although our study strongly indicated potential biomarkers for MF development, it is unclear whether they reliably predicted MF. This is because our results were obtained from the matrix files of different GEO datasets using independent background correction methods. More microarray samples and additional bioinformatics analyses are needed to comprehensively elucidate uncharacterized biomarkers. Second, further experiments should be conducted to validate the DEERSRGs acquired from our analysis. Examining the expression of the proteins encoded by more candidate DEERSRGs using molecular biology techniques, such as western blotting, reverse transcription-polymerase chain reaction, immunohistochemistry, and immunofluorescence assays, would also be useful. To investigate the function of target DEERSRGs at cell- and tissue-specific levels, a set of loss-and-gain-of-function experiments should be performed. However, overlapping molecular regulation by predicted genes during MF progression remains to be determined. The definitive evidence of the interaction between DEERSRG-encoded proteins requires further investigation by immunofluorescence, co-immunoprecipitation, and GST pull-down assays. Knowledge of the reciprocal mechanisms among the molecules that co-regulate the progression of MF remains incomplete, and it is necessary to test molecular activity using more assays related to PPI. Finally, more valuable prospective clinical studies are needed to bring these identified biomarkers into practice. These are beyond the scope of the current work but are potential directions for future investigations.

## Conclusions

ER stress is widely observed during MF and promotes the onset of autoimmune phenotypes that lead to DCM and are associated with poor prognosis. In this study, we identified seven hub genes, *AK1, ARPC3, GSN, KPNA2, PARP1, PFKL*, and *PRKC*, that might be involved in immune-related mechanisms.​ ​Enrichment analyses showed that DEERSRGs are closely related to biological pathways, including neuronal apoptosis, protein modification, oxidative stress response, glycolysis and gluconeogenesis, and NOD-like receptor signaling pathways. To the best of our knowledge, our work is the first to establish comprehensive regulatory networks for core genes associated with ER stress, which helps elucidate the biological mechanisms underlying DCM and MF. Our research paves the way for the future exploration of the role of ER stress-related genes and immune cell responses in MF, which might provide novel diagnostic strategies and therapeutic targets for patients with DCM.

## Data Availability

The data presented in this study are publicly available in the NCBI Gene Expression Omnibus (GEO) repository (GSE3585 and GSE42955).
