# Correction to: Integrative analysis of genes reveals endoplasmic reticulum stress-related immune responses involved in dilated cardiomyopathy with fibrosis

**DOI:** 10.1007/s10495-023-01881-x

**Published:** 2023-08-10

**Authors:** Wanpeng Li, Peiling Liu, Huilin Liu, Fuchun Zhang, Yicheng Fu

**Affiliations:** 1 Department of Cardiology, Gansu Provincial Hospital, 730000 Lanzhou, P.R. China; 2 Department of Rheumatology, First Affiliated Hospital of Zhengzhou University Zhengzhou, 450000 Henan, P.R. China; 3 Department of Geriatrics, Peking University Third Hospital, 100191 Beijing, P.R. China

**Apoptosis**

10.1007/s10495-023-01871-z

Unfortunately, In the original version of this article, the author names were listed incorrectly as

Wanpeng Li^1^ · Peiling Liu^2^ · Huilin Liu^3^ · Fuchun Zhang^4^ · Fu Yicheng^4^ · Yicheng Fu^4^

But, it should have been,

Wanpeng Li^1^, Peiling Liu^2^, Huilin Liu^3^ ,Fuchun Zhang^3^, Yicheng Fu^3^

In addition, the correct affiliation of authors will be as follows:

1. Department of Cardiology, Gansu Provincial Hospital, Lanzhou,730000, P.R China

2. Department of Rheumatology, First Affiliated Hospital of Zhengzhou University Zhengzhou, Henan, 450000, P.R. China

3. Department of Geriatrics, Peking University Third Hospital, Beijing, 100191, P.R China

The original article has been corrected.

